# Gut Epithelium of the Highly Toxic Ribbon Worm *Cephalothrix* cf. *simula* (Palaeonemertea, Nemertea) Contains Tetrodotoxin-Positive Bacterial Endosymbionts

**DOI:** 10.3390/toxins18030152

**Published:** 2026-03-23

**Authors:** Timur Yu. Magarlamov, Grigorii V. Malykin

**Affiliations:** A.V. Zhirmunsky National Scientific Center of Marine Biology, Far Eastern Branch, Russian Academy of Sciences, 690041 Vladivostok, Russia; gmalykin@imb.dvo.ru

**Keywords:** endosymbionts, symbiosome, TTX, tetrodotoxin-producing bacteria, ribbon worm, *Cephalothrix simula*

## Abstract

Tetrodotoxin (TTX), widely known as pufferfish venom, is a low-molecular-weight guanidinium neurotoxin. It can accumulate to extremely high concentrations in certain animals, including pufferfish, blue-ringed octopuses, flatworms, and nemerteans. However, the origin of TTX and the mechanisms that enable such extreme accumulation in these animals remain poorly understood. In this study, using confocal laser scanning microscopy combined with electron immunocytochemistry and ultrastructural analysis, we demonstrate the presence of TTX-positive bacteria associated with specialized cellular structures—type II phagosomes of gut enterocytes—in the highly toxic nemertean *Cephalothrix* cf. *simula*. We hypothesize that TTX production in *C*. cf. *simula* results from interactions between the nemertean host and its endosymbionts. These findings clarify the origin and accumulation of the toxin in nemerteans and have broader implications for other TTX-bearing species.

## 1. Introduction

Tetrodotoxin (TTX) is one of the most famous low-molecular-weight neurotoxins, accumulating at high concentrations in diverse animal taxa, including pufferfish [1,2,3], newts [4,5,6], flatworms [7,8,9,10], blue-ringed octopuses [11,12], and nemerteans [13,14,15,16]. In recent years, due to its strong and selective sodium channel–blocking activity, TTX has attracted considerable research interest as a promising anesthetic and analgesic alternative to opioid drugs [17,18]. However, its introduction into medical practice is significantly complicated by the limited understanding of its origin.

To date, direct evidence of TTX biosynthesis has been reported only for bacterial strains [19]. These putatively symbiotic or free-living bacteria are considered a potential source of TTX in highly toxic animals [19,20]. Recent studies on TTX-producing microflora have primarily focused on pufferfish [21,22,23,24,25], although TTX-producing bacteria have also been reported in the copepod *Pseudocaligus fugu* [26], the gastropod *Nassarius semiplicatus* [27], the goby *Yongeichthys criniger* [28], several newt species [29,30], and multiple nemerteans [31,32]. Most data are derived from bacterial cultures isolated either from whole-animal extracts [31,32] or from specific toxin-containing organs, such as glands, liver, skin, or intestine [21,22,23,24,33,34]. These findings suggest an association between tissue toxicity and the presence of TTX-producing bacteria. However, isolated cultures typically produce only trace amounts of TTX or lose this capacity after several passages [27], raising doubts about whether bacteria are the primary source of the toxin in highly toxic species. Consequently, the role of bacterial microflora in animal toxification remains unclear.

The detection of TTX-producing bacteria within the tissues of TTX-bearing animals using modern immunohistochemical methods, such as confocal laser scanning microscopy (CLSM) and electron microscopy with anti-TTX antibodies, provides a promising approach to clarifying their role in toxicity. However, previous immunohistochemical and ultrastructural studies of TTX-containing tissues in several animals [35,36,37] have not reported bacterial symbionts. Two immunoelectron microscopy studies, which provided detailed views of tissue ultrastructure and intracellular toxin localization, detected TTX-positive labeling exclusively in the animals’ own cells and subcellular structures [35,36]. In the pufferfish *Tetraodon nigroviridis*, TTX was localized in secretory (succiform) cells evenly distributed throughout the integument and associated with membrane-bound granules [35]. In the nemertean *Kulikovia alborastrata* (=*Lineus alborostratus*), TTX was localized in type I bacillary glandular cells of the body-wall cutis and in mucoid and pseudocnid-containing cells of the glandular epithelium of the proboscis [36]. Within glandular cells, TTX was detected in nuclear envelope, endoplasmic reticulum, and secretory granules.

In the present study, we examine TTX-containing tissues of the highly toxic nemertean *Cephalothrix* cf. *simula* for TTX-positive bacteria using CLSM with anti-TTX antibodies, combined with immunoelectron microscopy. Our results provide the first direct evidence of TTX-positive bacteria within the tissues of TTX-bearing animals and advance the understanding of the origin, distribution, and migration of TTX in ecosystems. These findings have important implications for the biotechnological production of TTX for biomedical applications, as well as for ecology and biosafety.

## 2. Results

### 2.1. Cephalic Gland

**Figure 1:**
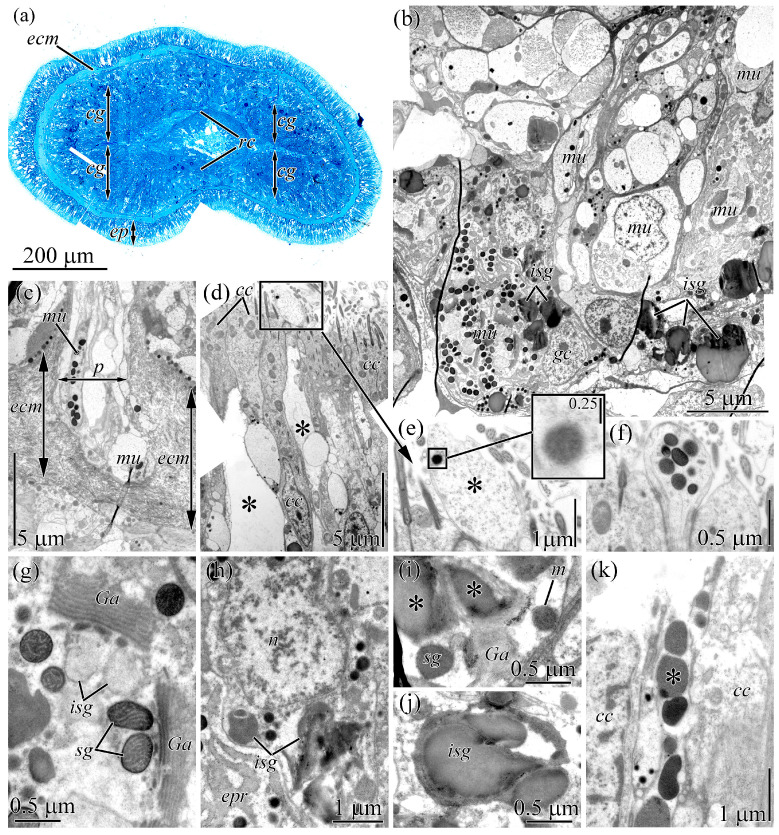
Light and electron micrographs of the cephalic gland of *Cephalothrix* cf. *simula*. (**a**) Panoramic view of the precerebral region. (**b**) Panoramic view of the central region of the cephalic gland. (**c**) Necks of cephalic glandular cells protruding through the subepidermal extracellular matrix. (**d**) Panoramic view of the integument showing a papilla of a mucous glandular cells (asterisks). (**e**) Papilla of a mucous glandular cell (asterisk) filled with fibrillary-granular content. High-magnification inset showing an intact granule. (**f**) Papilla of a mucous cell filled with intact granules. (**g**) Perinuclear cytoplasm of a mucous glandular cell. (**h**) Cell body of a granular cell. (**i**) Perinuclear cytoplasm of a granular cell with immature secretory granules (asterisks). (**j**) Immature secretory granules. (**k**) Excretory duct of granular cells containing secretory granules (asterisk) with homogeneous material with different electron density. Abbreviations: *cc*, ciliated cell; *cg*, cephalic gland; *ecm*, extracellular matrix; *ep*, integumentary epithelium; *epr*, endoplasmic reticulum; *Ga*, Golgi apparatus; *gc*, granular cell; *isg*, immature secretory granule; *m*, mitochondria; *mu*, mucous cell; *n*, nucleus; *p*, pore; *rc*, rhynchocoel; *sg*, secretory granule.

The cephalic gland occupies a significant part of the precerebral region of *C*. cf. *simula* (Figure 1a). Electron microscopy of the cephalic gland of *C*. cf. *simula* revealed two types of glandular cells, distinguished by granule shape and the appearance of their secretions: mucous and granular cells (Figure 1b).

The bodies of mucous cells are spherical (about 10 µm in diameter), with a single large nucleus positioned either centrally or toward the periphery (Figure 1b). A single cell neck extends from the proximal region of the cell body. The necks of mucous cells pass through pores in the subepidermal extracellular matrix (Figure 1c) and emerge on the surface of the integumentary epithelium as spherical papillae (Figure 1d–f). In the cell necks (Figure 1d) and papillae (Figure 1e), granules typically loosen to form a fibrillar-granular secretion, while only a few cell necks and papillae retain intact granules (Figure 1f). The perinuclear cytoplasm features 3–6 dictyosomes of the Golgi apparatus (GA), numerous mitochondria, and dilated cisternae of the endoplasmic reticulum (ER) (Figure 1g).

Granular cells have a spherical body (5–7 µm in diameter) with irregular, jagged borders with a nucleus positioned at the cell periphery (Figure 1b,h). The perinuclear cytoplasm harbors 2–4 dictyosomes of the GA and individual cisterns of the ER (Figure 1h,i). Large, irregularly shaped granules (immature granules) containing homogeneous material of medium electron density are associated with the trans pole of the dictyosomes (Figure 1j). In individual cells, immature secretory granules reach 4–5 µm in diameter. On semi-thin sections, these granules appear yellow. Granules in the excretory ducts are spherical (about 0.6 µm in diameter) and contain homogeneous material, with electron density ranging from medium to high (Figure 1k).

### 2.2. Integument

The cells of the integumentary epithelium of *C*. cf. *simula* can be broadly classified into two groups: ciliated and glandular cells [37].

**Figure 2:**
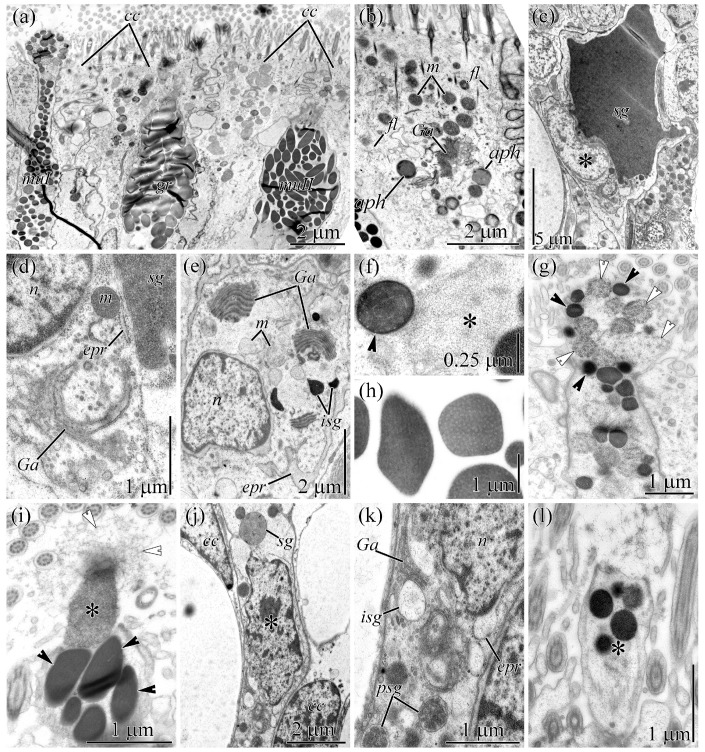
Transmission electron micrographs of transverse sections of the integument of *Cephalothrix* cf. *simula*. (**a**) Panoramic view of the integument. (**b**) Distal part of a ciliated cell. (**c**) Cell body of a serous cell (asterisk). (**d**) Perinuclear cytoplasm of a serous cell. (**e**) Cell body of a type I mucous cell. (**f**) Cell neck of a type I mucous cell showing intact (arrowhead) and destroyed (asterisk) secretory granules. (**g**) Papilla of a type I mucous cell with intact (black arrowheads) and partially destroyed (white arrowheads) secretory granules. (**h**) Secretory granules of a type II mucous cell. (**i**) Papilla of a type II mucous cell with intact (black arrowheads) and partially destroyed (asterisk) secretory granules. The partially destroyed granule (asterisk) is surrounded by fibrillar material (white arrowheads). (**j**) Cell body of a granular cell (asterisk). (**k**) Perinuclear cytoplasm of a granular cell. (**l**) Papilla (asterisk) of a granular cell with intact secretory granules. Abbreviations: *aph*, autophagosome; *cc*, ciliated cell; *epr*, endoplasmic reticulum; *fl*, intermediate filaments; *Ga*, Golgi apparatus; *gr*, granular cell; *isg*, immature secretory granule; *m*, mitochondria; *muI/II*, type I/II mucous cell; *n*, nucleus; *psg*, premature secretory granule; *sg*, secretory granule.

Ciliated cells are the most abundant cell type in the integument. Their bodies are strongly elongated and funnel-shaped, with a widened apical part that tapers toward the proximal end, gradually continuing into a narrow stalk (Figure 2a).

The apical surface bears numerous cilia and microvilli. The cytoplasm contains numerous mitochondria and autophagosomes, a well-developed dictyosome of the GA, individual cisternae of the ER, and an extensive network of intermediate filaments (Figure 2b).

Electron microscopy revealed four types of glandular cells: serous cells, two types of mucous cells (type I and II), and granular cells.

Serous cells vary in shape from goblet-like, with a narrowed apical portion, to lanceolate (Figure 2c). Their nuclei are round and positioned in the lower half of the cell, typically just beneath the granule. The perinuclear cytoplasm and basal region contain numerous mitochondria, dictyosomes of GA, cisternae of the ER, and numerous small vesicles up to 0.1 µm in diameter (Figure 2d). The majority of the cytoplasm is occupied by a single large secretory granule filled with homogeneous, electron-dense material.

Type I and type II mucous cells are club-shaped, with an expanded basal body from which a narrow neck extends, widening significantly toward the apical surface (Figure 2a). The cell body harbors 2–4 dictyosomes of the GA, mitochondria, individual cisternae of the ER, and secretory granules at various stages of maturation. Type I and type II mucous cells differ in the shape, size, and internal structure of their glandular granules (Figure 2e). In type I mucous cells, the glandular granules are spherical (up to 0.5 μm in diameter) and contain curved fibrils of low electron density embedded in a homogenous, electron-dense matrix (Figure 2f). Some granules in the cell necks (Figure 2f, asterisk) and papillae (Figure 2g) appear loosened and consist of thick fibrils (25–60 nm in diameter) embedded in a loosely packed fibrous matrix. In type II mucous cells, the glandular granules are oval with angular outlines, reaching up to 2 μm in length and about 0.5 μm in diameter (Figure 2a). Their contents comprise parallel fibrils of low electron density embedded in a homogenous, electron-dense matrix (Figure 2h). Granules in the cell necks and papillae are intact. Upon release from the papilla, the granules lose their structural integrity and form a single fibrillar mass (Figure 2i, white arrowheads).

Granular cells are goblet-shaped, with an expanded cell body (Figure 2a,j) that tapers into a narrow apical neck terminating in an expanded papilla (Figure 2l). The perinuclear cytoplasm harbors dilated cisternae of the ER, one to two dictyosomes of the GA, and numerous secretory granules at different stages of maturation (Figure 2k). The cytoplasm of the cell necks and papillae is filled with spherical secretory granules measuring 0.4–0.7 µm in diameter (Figure 2a,l). The granules contain homogeneous material of medium to high electron density.

### 2.3. Proboscis

Ultrastructural analysis of the proboscis epithelium revealed a single type of epithelial cells (supportive cells) and three types of glandular cells.

**Figure 3:**
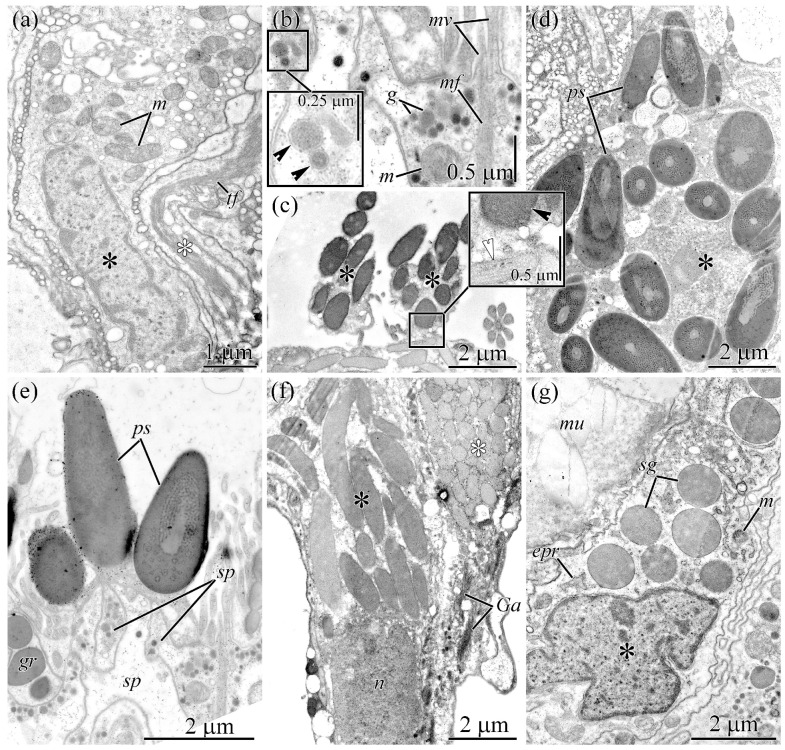
Transmission electron micrographs of transverse sections of the glandular epithelium of the proboscis of *Cephalothrix* cf. *simula*. (**a**) Cell body of a supportive cell (black asterisk). The white asterisk indicates the cell “leg”. (**b**) Distal region of a supportive cell. High-magnification inset shows membrane-bound granules (arrowheads). (**c**) Papillae of mucous cells (asterisks). High-magnification inset shows granules with homogenous (black arrowhead) and fibrillar (white arrowhead) contents. (**d**) Cell body of a pseudocnid-forming cell (asterisk). (**e**) Pseudocnidae resting on supportive cells. (**f**) Proximal region of the glandular epithelium. Some mucous cells contain secretory granules with densely packed fibrous material (black asterisk), whereas others contain moderately packed fibrous material (white asterisk). (**g**) Cell body of a granular cell (asterisk). Abbreviations: *epr*, endoplasmic reticulum; *g*, granule; *Ga*, Golgi apparatus; *gr*, granular cell; *m*, mitochondria; *mv*, microvilli; *mf*, microfilaments; *mu*, mucous cell; *ps*, pseudocnida; sg, mature secretory granule; *sp*, supportive cell; *tf*, tonofilaments.

Supporting cells are thin, highly branched cells that extend between the glandular cell bodies (Figure 3a).

They possess an elongated, oval nucleus located in the basal region of the cytoplasm (Figure 3b). The cytoplasm harbors numerous mitochondria, cisternae of the ER, and dictyosomes of the GA, as well as round, membrane-bound granules (0.1–0.2 µm in diameter) filled with heterogeneous material of moderate or high electron density (Figure 3b).

Three types of glandular cells were distinguished by the shape and content of their secretory granules: pseudocnid-containing cells, mucous cells, and granular cells.

Pseudocnid-containing cells have unique secretory granules, called pseudocnidae, in the cytoplasm (Figure 3d). Two types of pseudocnid-containing cells—small and large—were identified, differing only in the size and internal structure of their pseudocnidae. The cell bodies contain a large, centrally located nucleus, numerous pseudocnidae at various stages of maturation, mitochondria, and cisternae of the ER (Figure 3d). Mature pseudocnidae are released onto the apical surface of the supporting cells, where they form small clusters of 3–5 granules each (Figure 3c).

Mucous cells are oval and have an oval nucleus located in the basal region of the cell (Figure 3f). The perinuclear cytoplasm features numerous cisternae of the ER and mitochondria, while the remaining cytoplasm is occupied by secretory granules at various stages of maturation (Figure 3f). Immature secretory granules are oval, measuring up to 1.7 µm in length 0.6 µm in width, and contain loose fibrillary material. During maturation, the granules elongate into a highly bacillary or rod-shaped form (Figure 3d), reaching approximately 6 µm in length and 1 µm in width, while their interior consists of densely packed, parallel fibrils.

Granular cells are oval, with an irregularly shaped nucleus located in the central region of the cytoplasm (Figure 3g). Their cytoplasm is rich in large mitochondria, cisternae of the ER, and dictyosomes of the GA. The central and apical regions of the cells are occupied by rounded granules approximately 1.3 µm in diameter, filled with homogeneous material of moderate electron density (Figure 3g).

### 2.4. Gut

**Figure 4:**
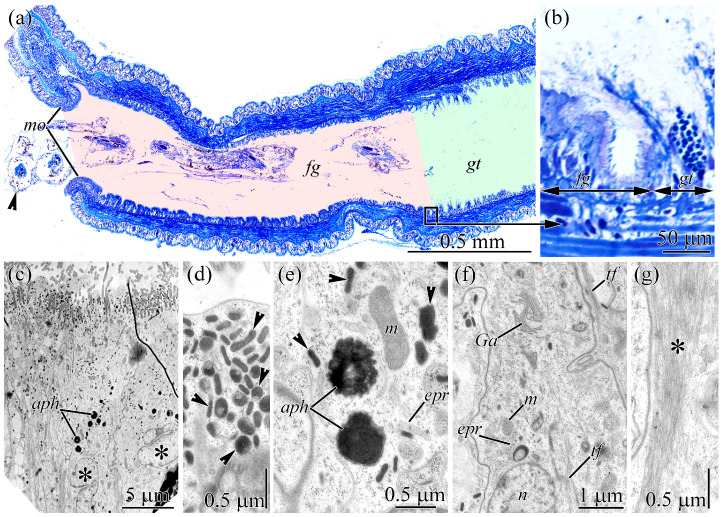
Light and transmission electron micrographs of the digestive system of *Cephalothrix* cf. *simula*. (**a**) Panoramic view of the anterior end of the worm. Arrowheads show diet items eaten by ribbon worm. (**b**) Transition between the foregut and the gut. (**c**) Panoramic view of the foregut epithelium showing non-phagocytic enterocytes (asterisks). (**d**) Apical region of non-phagocytic enterocytes with rod-shaped, membrane-bound structures (arrowheads). (**e**) Central region of non-phagocytic enterocytes showing singly scattered rod-shaped, membrane-bound structures (arrowheads). (**f**) Cell body of a non-phagocytic enterocyte. (**g**) Leg of a non-phagocytic enterocyte filled with bundles of tonofilaments (asterisk). Abbreviations: *aph*, autophagosomes; *epr*, endoplasmic reticulum; *fg*, foregut; *gt*, gut; *Ga*, Golgi apparatus; *m*, mitochondria; *mo*, mouth; *n*, nucleus; *tf*, tonofilaments.

The digestive tract of *C*. cf. *simula* is lined with a single-layered, pseudostratified epithelium. Morphologically, it can be divided into two sections: the foregut and the gut (Figure 4a).

The foregut extends from the mouth opening for approximately 1 cm and terminates in an epithelial groove that is clearly visible in semi-thin sections (Figure 4a). The gut follows the foregut directly (Figure 4b) and ends at the anus at the posterior tip of the worm. The epithelium of the digestive tract is composed of glandular cells and enterocytes. Since the glandular cells have been characterized previously [38], this study focuses only on the fine structure of enterocytes.

In the foregut, only non-phagocytic enterocytes were observed. These cells are elongated and rectangular, with expanded apical and middle regions and a narrowed basal region (leg) (Figure 4c). Their apical surface is densely covered with cilia and microvilli, which are evenly distributed. Cross-sections of individual enterocytes reveal 5–11 cilia and 5–10 microvilli. In the premembrane layer beneath the apical cytoplasm, numerous evenly distributed, oval, rod-shaped, membrane-bound structures are present, measuring up to 0.3 µm in length and about 0.1 µm in width (Figure 4d). They contain densely packed, fine-grained material. Individual rod-shaped structures are also observed in the middle region of the cytoplasm (Figure 4e). The cell nucleus is oval and situated centrally (Figure 4c). The cytoplasm features well-developed ER and GA, with mitochondria showing well-developed cristae distributed evenly throughout (Figure 4e,f). The apical and middle regions of the cytoplasm contain spherical granules (up to 1 µm in diameter) with heterogeneous contents (autophagosomes) (Figure 4c,e). The basal legs of the cells extend between the bodies of adjacent cells (Figure 4g) and attach to the underlying membrane of extracellular matrix (ECM). Their cytoplasm harbors a plexus of tonofilaments oriented along the apical–basal axis of the cells.

**Figure 5:**
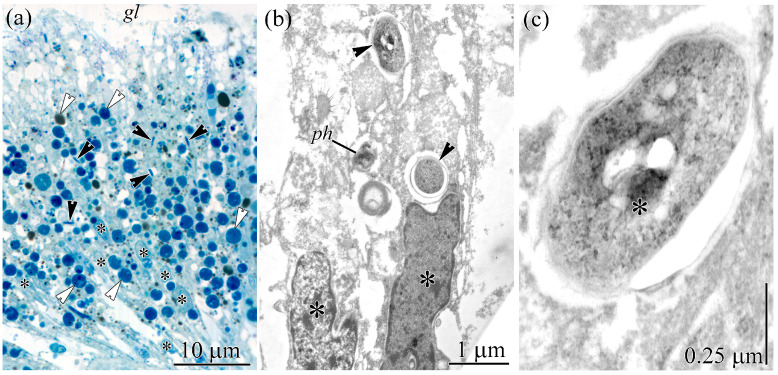
Transmission electron micrographs of the gut epithelium of *Cephalothrix* cf. *simula*. (**a**) Panoramic view of the gut epithelium. Enterocytes (asterisks) harbor spherical (type I; white arrowheads) and rod-shaped (type II; black arrowheads) phagosomes. (**b**) Middle region of the gut epithelium showing enterocytes (asterisks) with type II phagosomes (arrowheads). (**c**) Enterocyte type II phagosomes containing bacteria (asterisk). Abbreviations: *gl*, gut lumen; *ph*, type I phagosome.

The gut is composed exclusively of phagocytic enterocytes, elongated, rectangular cells with an expanded apical region that gradually tapers to a narrow basal portion (Figure 5a).

The apical surface is densely covered with evenly distributed microvilli and cilia. Cross-sections reveal approximately 5–7 cilia and 3–5 microvilli per cell. A large, round or oval nucleus occupies the central region of the cell (Figure 5a). The central and apical cytoplasm houses cisternae of the ER, dictyosomes of the GA, a small number of mitochondria, and numerous phagosomes evenly distributed throughout (Figure 5a,b). Two types of phagosomes can be distinguished based on their shape and contents. Type I phagosomes are spherical, 1–5 μm in diameter, and contain heterogeneous material (Figure 5a,b). Type II phagosomes are rod-shaped, approximately 4 μm long and up to 1 μm wide (Figure 5a), each containing one to three bacteria arranged in a chain (Figure 5b,c). The bacteria are bacillary, measuring up to 3.8 µm in length and 0.8 µm in width, and possess a two-layered cell wall typical of Gram-negative bacteria (Figure 5c). Their central (nuclear) region exhibits fibrillar and granular material, while the peripheral cytoplasm consists of finely granular material interspersed with fine fibrils. Spherical vacuoles, 20–80 nm in diameter, are evenly distributed throughout the cytoplasm, with 5–20 vacuoles filled with electron-lucent material per section (Figure 5c).

**Figure 6:**
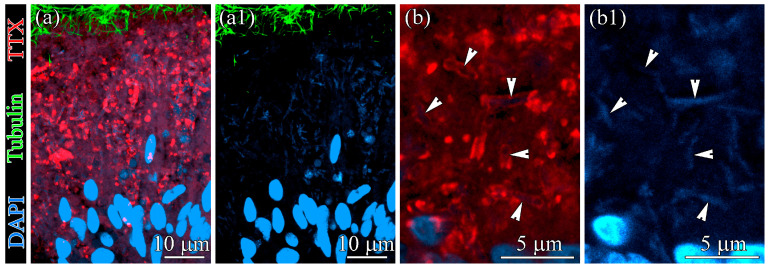
Confocal laser scanning micrographs of the gut epithelium of *Cephalothrix* cf. *simula* labeled with antibodies against tetrodotoxin (TTX) and acetylated α-tubulin. (**a**) Overview of the gut epithelium. (**a1**) Substack of DAPI signal and acetylated α-tubulin-like-immunoreactivity. (**b**) Substack of proximal region of the gut epithelium. Arrowheads indicate TTX-positive bacteria. (**b1**) Substack of DAPI signal. Arrowheads indicate nucleoids of TTX-positive bacteria.

In the study by Malykin et al. [37], TTX-positive labeling was detected in glandular cells of the foregut and in enterocytes of the gut. In the present study, we examined the distribution and characteristics of TTX-positive structures in gut enterocytes using CLSM with anti-TTX antibodies. Although the cytoplasm of enterocytes showed weak fluorescence, intensely fluorescent TTX-positive structures were clearly observed (Figure 6a).

These structures were rod-shaped, measuring up to 4 μm in length and 1 μm in width, and exhibited labeling along their outer perimeter (Figure 6b). Their interiors showed uniform DAPI-positive labeling. The highest density of rod-shaped structures was found in the apical and central regions of enterocytes. The number of such structures per 1 cm^2^ section of the anterior, middle, and posterior regions of the worm’s gut was 28.6 ± 21.1 (mean ± standard deviation, n = 10), 111.7 ± 29.4 (n = 10), and 70.9 ± 18.3 (n = 10), respectively. Statistical Kruskal–Wallis H test showed that the number of TTX-positive rod-shaped structures in the different regions of the worm’s gut were statistically different (*p*-value < 0.05).

**Figure 7:**
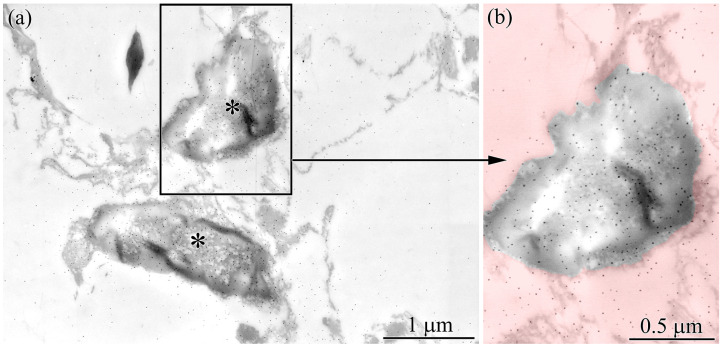
Immunoelectron micrographs of the gut epithelium of *Cephalothrix* cf. *simula*. (**a**) Apical region of an enterocyte showing endosymbiotic bacteria (asterisks). (**b**) High-magnification view of an endosymbiotic bacterium. Red indicates the cytoplasm of the enterocyte.

The gut was examined using electron immunocytochemistry, and TTX-positive labeling was quantified in the resulting images. Labeling was observed in the cytoplasm and within type II phagosomes (Figure 7a,b).

Quantification of particles in a 5.7 × 4 µm (22.8 µm^2^) image showed that type II phagosomes contained, on average, 3.1 times more colloidal gold (n = 5) than the surrounding cytoplasm.

## 3. Discussion

To date, highly toxic nemerteans of the *Cephalothrix simula* species complex (comprising *Cephalothrix simula*, *C*. cf. *simula*, and *Cephalothrix mokievskii*) rank among the most thoroughly studied animals with respect to TTX localization in cells and tissues. Previous studies have used immunohistochemistry and light microscopy to examine TTX distribution in toxic organs and their tissue structure [37,39], while the foregut of *C*. cf. *simula* has been examined in greater detail using electron microscopy [38]. Although TTX has been detected in cells and organs involved in hunting and defense, no associated bacteria have been identified. In this study, we used transmission electron microscopy (TEM) to examine and characterize the ultrastructure of cells in TTX-containing organs of *C*. cf. *simula*, including the integument, cephalic gland, proboscis, and gut. Our analysis not only clarifies the organization of TTX-positive structures but also reveals a putative source of the toxin: symbiotic bacteria in the gut epithelium.

Five cell types were identified in the integument of *C*. cf. *simula*: ciliated cells, serous cells, mucous cells of types I and II, and granular cells. Tanu et al. [39] reported intense TTX-positive labeling in bacillary cells, which correspond to the granular cells identified in this study. Similarly, Malykin et al. [37] observed such labeling in serous cells, type III granular cells (also corresponding to granular cells here), and ciliated cells. According to Malykin et al. [37], intense TTX-positive labeling in the excretory ducts and papillae of serous cells and type III granular cell was directly associated with secretion. In addition, strong labeling was found in the perinuclear cytoplasm of these cells, where it was associated with tubular structures identified as thin sinuous filaments (rough endoplasmic reticulum). Our ultrastructural analysis shows that, in serous and granular cells, the perinuclear cytoplasm contains only components of the heterosynthetic apparatus and immature secretory granules, whereas the cell necks and papillae contain exclusively mature secretory granules. No bacteria or structures resembling bacterial cells were detected.

We detected no bacterial symbionts or bacteria-like structures in the cells of the cephalic gland or in the glandular epithelium of the proboscis. Detailed ultrastructural analysis refined the classification of glandular cell in both organs, demonstrating that granular cells of types I and II in the cephalic gland and proboscis (according to Malykin et al. [37]) represent a single cell type, here referred to as granular cells.

Several structures in the digestive system of *C*. cf. *simula* exhibit TTX-positive labeling. Tanu et al. [39] reported TTX immunostaining in vesicles scattered along the foregut wall. Subsequent analyses using CLSM with anti-TTX antibodies, combined with TEM, revealed intense labeling in the granules of glandular cells of types I and V, as well as in rod-shaped structures located in the premembranous apical cytoplasm of ciliated cells in the foregut [38]. According to the present study, these rod-shaped structures are membranous organelles that likely function in prey immobilization within the foregut. Interestingly, in *C*. cf. *simula* larvae, clusters of similar organelles have been observed in the apical cytoplasm of cells in the provisional epidermis and around the mouth opening [40], suggesting an important functional role throughout ontogeny.

In a study of TTX localization in the gut of *C*. cf. *simula* [37], numerous phagosomes were observed in the cytoplasm of phagocytic enterocytes. The authors distinguished two types of phagosomes: TTX-positive phagosomes (hereafter type II) and phagosomes lacking TTX labeling (hereafter type I). We found that type II phagosomes contain TTX-positive bacteria (Figure 6 and Figure 7), whereas type I phagosomes contain only heterogeneous material representing digested food (Figure 5a,b). The presence of TTX-positive bacteria within type II phagosomes explains why only this type exhibits luminescence in the gut of nemerteans.

Structures resembling type II phagosomes that house symbiotic bacteria are referred to as “symbiosomes” in the literature [41]. These organelles shelter, nourish, and support microorganisms that benefit their host. Symbiotic bacteria may provide nutrients or recycle waste, particularly nitrogenous compounds [42,43], and can also supply growth factors or chemical defenses [43,44]. The interaction between type II phagosomes and resident bacteria may thus represent a potential source of TTX in nemerteans. Notably, ultrastructural studies have not detected bacteria in the gut of *C*. cf. *simula* larvae up to day 42 of development [40]. Consequently, further studies of juvenile specimens are required to determine when symbiosis and the formation of type II phagosomes are established.

The source of TTX in animals remains a matter of debate. Its occurrence across diverse eukaryotic lineages has led to the hypothesis that symbiotic bacteria within the host microflora produce the toxin [19,45]. Recent metagenomic analyses of microflora in TTX-bearing pufferfish [46] and newts [30] further support the role of symbiotic bacteria in animal toxicity. However, because the extremely high TTX levels observed in many species cannot be fully explained by bacterial production alone, it has been suggested that the toxin accumulates through the food chain [47]. This hypothesis is supported by experiments on pufferfish, which showed that individuals kept in captivity lose their toxicity when deprived of TTX-containing food, but can regain it when provided with an appropriate toxin-containing diet [47,48]. Moreover, highly toxic flatworms *Planocera multitentaculata* [49,50] and *C. simula* [51] may serve as natural sources of TTX for wild pufferfish. Experiments on TTX-bearing newts maintained on a TTX-free diet have yielded conflicting results, with some studies reporting persistent toxicity [52,53] and others showing a gradual decline [54].

**Figure 8:**
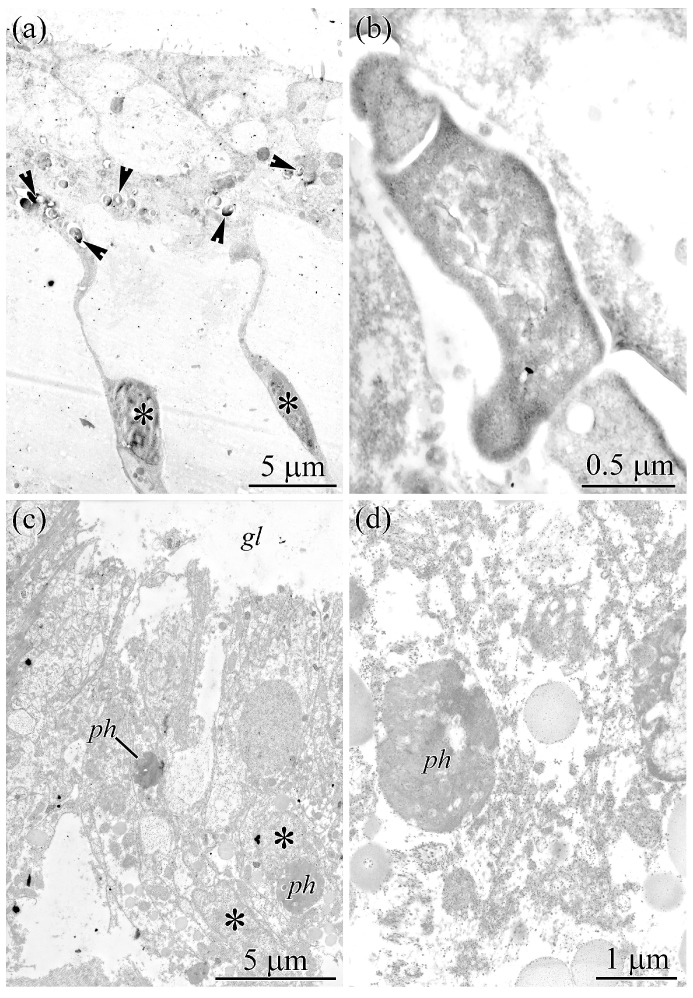
Transmission electron micrographs of the gut epithelium of *Cephalothrix* spp. (**a**) *Cephalothrix mokievskii*, gut epithelium composed of enterocytes (asterisks). Arrowheads indicate type II phagosomes containing bacterial cells. (**b**) *C. mokievskii*, enterocyte type II phagosomes with bacteria. (**c**) *Cephalothrix filiformis* sensu Iwata [59], gut epithelium composed of enterocytes (asterisks). (**d**) *C. filiformis* sensu Iwata [59], central region of an enterocyte showing a phagosome. Abbreviations: *gl*, gut lumen; *ph*, phagosome.

Although food-chain accumulation may partially explain high TTX levels in toxic vertebrates, a dietary origin has not been confirmed for toxic invertebrates. In *P*. *multitentaculata*, no uptake of TTX from major dietary items has been demonstrated [55]. Similarly, in *C*. cf. *simula*, no correlation between diet and toxicity has been observed [56], and screening of numerous potential food sources has revealed no significant TTX levels [57]. Notably, nemerteans of the *Cephalothrix simula* species complex are able to maintain their toxicity when kept in captivity on a TTX-free diet [58]. Thus, TTX-positive symbiotic bacteria in the gut of *C*. cf. *simula* support a bacterial origin of TTX. To further test this hypothesis, we examined intestinal tissues of the highly toxic *C*. *mokievskii* (a member of the *Cephalothrix simula* species complex) and the non-toxic *Cephalothrix filiformis* sensu Iwata [59]. Enterocytes in *C*. *mokievskii* contained symbiosomes (type II phagosomes) with bacteria (Figure 8a,b), similar to those observed in *C*. cf. *simula*. In contrast, enterocytes of *C*. *filiformis* sensu Iwata [59] contained only digestive phagosomes filled with heterogeneous material (Figure 8c,d), lacking intact bacterial cells. Notably, TTX-positive bacteria were consistently observed in all examined individuals of toxic nemerteans, although the limited sample size does not exclude potential biological variability in their abundance.

It should be noted that accumulation of TTX through the food chain cannot be entirely excluded [57], and it remains possible that the bacteria identified in this study may also accumulate TTX derived from dietary sources. Nevertheless, the data obtained here can support a bacterial contribution to TTX origin and open new perspectives for investigating tissues of other TTX-bearing invertebrates for TTX-positive microorganisms, thereby advancing understanding of toxin origin.

## 4. Conclusions

1. In this study, we report the presence of TTX-positive intracellular symbiotic bacteria within the tissues of TTX-bearing animals. Because all members of the closely related *Cephalothrix simula* species complex contain high concentrations of TTX, it is reasonable to hypothesize that symbiotic TTX-positive microorganisms are widespread throughout this group. This hypothesis will be tested in future studies using ultrastructural analyses of the gut in *C*. *simula* and other highly toxic nemerteans.
2. Given that TTX is considered a promising anesthetic and analgesic agent for the treatment of various types of pain, future research should focus on isolating, genetically identifying, and cultivating the endosymbiotic bacteria detected in this study under controlled conditions. Subsequent analyses should assess TTX production in culture and aim to identify the genes responsible for toxin biosynthesis, which remain unknown. Such studies would clarify whether these endosymbionts directly produce TTX and could facilitate the development of recombinant TTX production technologies for biomedical applications.
3. Based on our findings, we hypothesize that *C*. cf. *simula* forms symbiotic associations with specific bacterial strains and may stimulate TTX production in these partners. In this context, nemerteans may represent a key link in the transfer of TTX to higher trophic levels, including predators such as pufferfish. Intracellular TTX synthesis by symbiotic microflora may also occur in other TTX-bearing animals (e.g., flatworms and newts), highlighting the need to search for comparable symbionts across diverse taxa.

## 5. Materials and Methods

### 5.1. Collection of Nemerteans

Four individuals of *C*. cf. *simula* were collected in Spokoynaya Bay, Sea of Japan (42.7° N, 133.2° E), in August 2020 and June 2024 by Grigorii V. Malykin, among the rhizoids of the brown alga *Saccharina japonica* at depths of 0.5–2 m. Three individuals of *C*. *mokievskii* were collected in August 2024 from rhizoids of the biennial brown alga *Saccharina* sp. in Aniva Bay, Sea of Okhotsk (46.2° N, 142.5° E) by Timur Yu. Magarlamov. Two individuals of *C*. *filiformis* sensu Iwata [59] were collected in August 2015 by Alexei V. Chernyshev under stones in the littoral zone of Ussuri Bay, Sea of Japan (43.1° N, 132° E). All animals were collected from not-protected areas that did not require research permits. All experiments with animals were carried out in accordance with ARRIVE guidelines.

After collection, animals were maintained individually in tanks containing filtered, aerated seawater (17 °C) for 1–3 days prior to experimental procedures. Seawater was filtered through hydrophobic polyvinylidene fluoride membrane filters with pore size 0.45 µm (Merck Millipore, Burlington, MA, USA). Before invasive procedures, nemerteans were anesthetized in 7% MgCl_2_. Prior to fixation, specimens were rinsed three times in filtered seawater. Tetrodotoxin and its analogues (TTXs) profiles and concentrations in *C*. cf. *simula* and *C*. *mokievskii* tissues were analyzed previously using high-performance liquid chromatography–tandem mass spectrometry (HPLC-MS/MS) [37,58]. The absence of TTXs in *C*. *filiformis* was confirmed using the same protocol (see Malykin et al. [58]).

Preliminary identification of *C*. cf. *simula* and *C*. *mokievskii* based on morphological characteristics was performed by Alexei V. Chernyshev and Grigorii V. Malykin. Preliminary identification of *C*. *filiformis* sensu Iwata [59] was performed based on morphological characteristics by Alexei V. Chernyshev. Molecular identification was subsequently carried out. Molecular data for *C*. *filiformis* sensu Iwata [59] were reported by Chernyshev and Polyakova [60], with sequences deposited in the DDBJ/ENA/GenBank databases under accession numbers MW136194, MW136199, MW136152, MW118019, MW118028. Molecular identification of *C*. cf. *simula* and *C*. *mokievskii* was described by Malykin et al. [58], with sequences deposited under accession numbers PV984387–PV984388.

### 5.2. Ultrastructural Analysis

Specimens of *C*. cf. *simula, C*. *mokievskii,* and *C*. *filiformis* sensu Iwata [59] were fixed in 2.5% glutaraldehyde in cacodylate buffer, post-fixed in 1% osmium tetroxide, and dehydrated through a graded series of ethanol and acetone. Samples were embedded in Epon-Araldite resin (EMS, Hatfield, PA, USA). Semithin (1 µm) and ultrathin (60 nm) sections were prepared using an Ultracut E ultramicrotome (Reichert-Leica Biosystems, Wetzlar, Germany). Semithin sections were stained with methylene blue (Sigma-Aldrich, St. Louis, MO, USA) and examined under a Zeiss Axio Imager Z2 microscope (Carl Zeiss, Jena, Germany). Ultrathin sections were stained with 1% uranyl acetate and 0.35% lead citrate and observed using a Zeiss Libra 120 transmission electron microscope (Carl Zeiss, Jena, Germany). The original, unadjusted image files are available at https://doi.org/10.6084/m9.figshare.31337611.

### 5.3. Confocal Laser Scanning Microscopy

Tissue localization of TTX was examined in *C*. cf. *simula* using CLSM (Carl Zeiss, Jena, Germany). Tissue samples (~2 mm segments from the anterior, middle, and posterior body regions) were fixed in 4% paraformaldehyde in phosphate-buffered saline (PBS, pH 7.8). Specimens were then embedded in 20% sucrose and sectioned at 10 µm using a Thermo HM 560 cryotome (Thermo Fisher Scientific, Waltham, MA, USA). Sections were incubated for 30 min in blocking solution (10% bovine serum albumin (BSA) and 10% normal goat serum in PBS). For TTX labeling, sections were incubated with rabbit polyclonal anti-TTX antibody (Genetex, Irvine, CA, USA) diluted 1:25 in PBS containing 10% BSA. Tubulin-immunoreactive structures were visualized using mouse polyclonal anti-acetyl α-tubulin antibody (Sigma-Aldrich, St. Louis, MO, USA) diluted 1:1000 in PBS with 10% BSA. Sections were incubated in primary antibodies for 48 h at 4 °C, then washed in PBS with 0.05% Tween-20 (TBST, Sigma-Aldrich, MO, USA). Sections were then incubated for 24 h at 4 °C with goat anti-mouse Alexa Fluor 488 (1:500, Thermo Fisher Scientific, MA, USA) and goat anti-rabbit Alexa Fluor 647 (1:500, Thermo Fisher Scientific, MA, USA), followed by nuclear staining with 4′,6-diamidino-2-phenylindole (DAPI, Thermo Fisher Scientific, MA, USA). Stained sections were mounted in Mowiol 4-88 (Sigma-Aldrich, MO, USA) and examined using a Zeiss LSM-780 confocal microscope (Carl Zeiss, Jena, Germany). Cryosections of *C*. cf. *simula* incubated with non-immune rabbit serum in PBS with 10% BSA, instead of the primary antibody, were used as negative controls. These sections were processed identically to experimental samples, and no positive TTX-labelling was detected (Supplementary File S1). To confirm antibody specificity, cryosections of *C*. cf. *simula* were incubated with primary anti-TTX antibody that had been preabsorbed for 2 h at 4 °C with a *C*. cf. *simula* extract containing a known concentration of TTX (1000 ng/mL, diluted 1:10 *v*/*v* in antibody solution) and processed in the same manner as experimental samples (Supplementary File S1). All specimens were imaged using the 647 nm channel to rule out aberrant autofluorescence.

### 5.4. Immunoelectron Microscopy

Intracellular localization of TTX in *C*. cf. *simula* was examined using immunoelectron microscopy. Tissue samples (~2 mm segments from the anterior, middle, and posterior body regions) were fixed in 4% formaldehyde in PBS for 1 h, rinsed in PBS for 2 h, dehydrated in graded ethanol and acetone, and embedded in LR White resin (EMS, Hatfield, PA, USA). Ultrathin sections (60 nm) were prepared using an Ultracut E ultramicrotome (Leica Biosystems, Wetzlar, Germany). Sections were incubated for 30 min in blocking solution (10% BSA and 10% normal goat serum in PBS), followed by incubation with polyclonal rabbit anti-TTX primary antibodies (Thermo Fisher Scientific, MA, USA) diluted 1:25 in PBS containing 10% BSA. Primary antibody incubation was carried out at 4 °C for 24 h, followed by washing in PBS at 4 °C for 24 h. Sections were then incubated at 4 °C for 24 h with goat anti-rabbit IgG conjugated to gold nanoparticles (Thermo Fisher Scientific, MA, USA) diluted 1:50 in PBS containing 1% BSA. Sections were then were treated with the LI Silver Enhancement Kit (convenient, light-insensitive silver enhancement system) (Thermo Fisher Scientific, MA, USA) for 30 min, following the manufacturer’s protocol. After that, the sections were stained with 1% uranyl acetate.

Samples were examined using a Zeiss Libra 120 transmission electron microscope (Carl Zeiss, Jena, Germany). To assess the specificity of TTX labeling, the density of gold nanoparticles associated with bacterial cells was compared with that in surrounding nemertean tissues.

## Data Availability

The original contributions presented in this study are included in the article/Supplementary Materials and loaded to Figshare repository (https://doi.org/10.6084/m9.figshare.31337611). Further inquiries can be directed to the corresponding author.

## Supplementary Materials

The following supporting information can be downloaded at: https://www.mdpi.com/article/10.3390/toxins18030152/s1, Figure S1. Tetrodotoxin-like immunoreactivity in the anterior part of *Cephalothrix* cf. *simula*.

## Abbreviations

The following abbreviations are used in this manuscript:

TTX	Tetrodotoxin
CLSM	Confocal laser scanning microscopy
GA	Golgi apparatus
ER	Endoplasmic reticulum
ECM	Extracellular matrix
TEM	Transmission electron microscopy
PVDF	Hydrophobic polyvinylidene fluoride
HPLC-MS/MS	High-performance liquid chromatography with tandem mass-spectrometry
