# Supplementary material for: Gut Epithelium of the Highly Toxic Ribbon Worm Cephalothrix cf. simula (Palaeonemertea, Nemertea) Contains Tetrodotoxin-Positive Bacterial Endosymbionts
Source: Toxins (Basel). 2026 Mar 23;18(3):152. doi: 10.3390/toxins18030152 (PMC13030763; doi:10.3390/toxins18030152)
Supplement: Supplementary file 1 [file toxins-18-00152-s001.zip › toxins-4176811-supplementary.pdf]

Supplementary Materials: Gut epithelium of the highly toxic ribbon worm Cephalothrix cf. simula (Palaeonemertea, Nemertea) contains tetrodotox-in-positive bacterial endosymbionts

Timur Yu. Magarlamov * and Grigorii V. Malykin

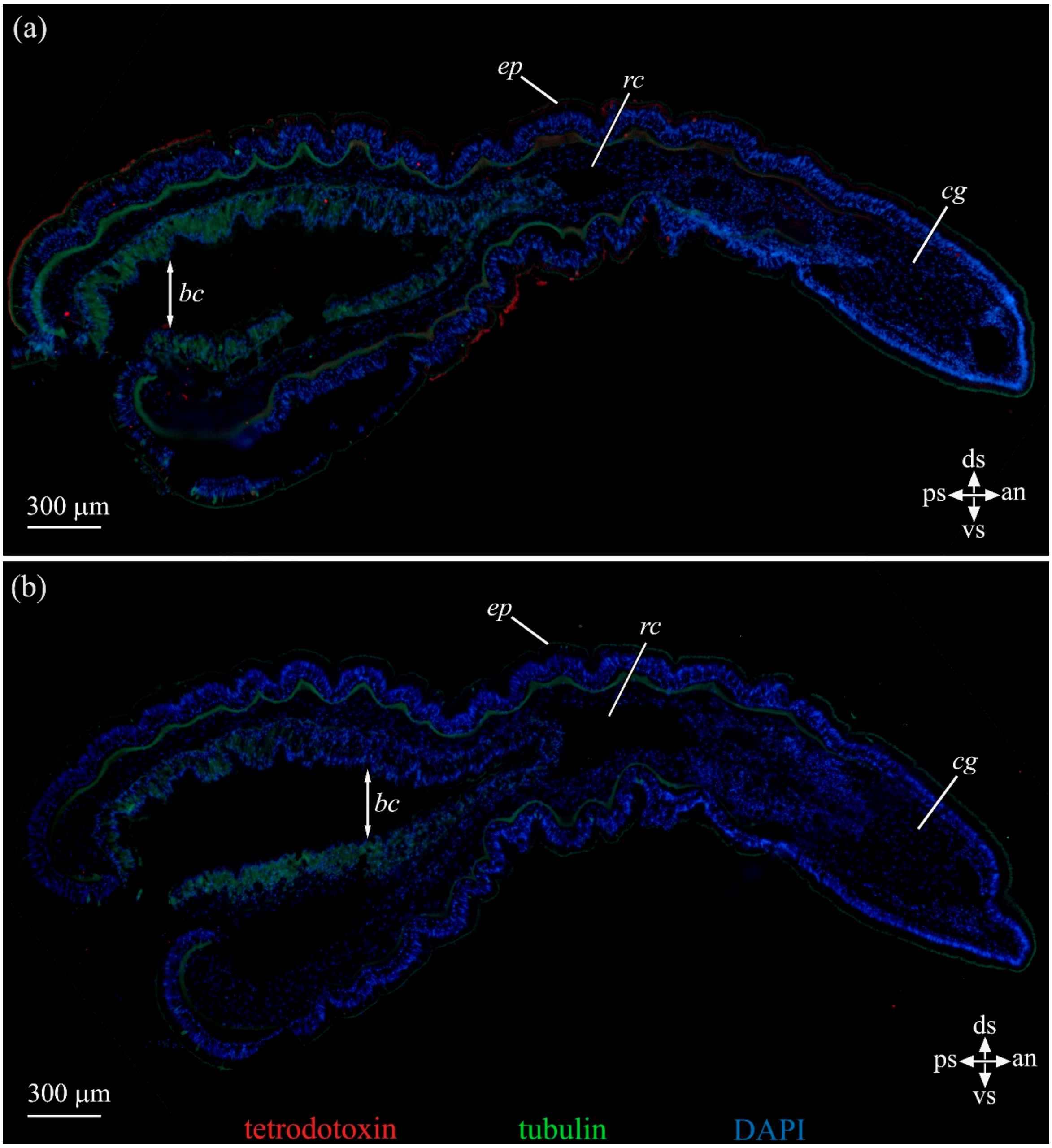

Figure S1. Tetrodotoxin-like immunoreactivity in the anterior part of *Cephalothrix* cf. *simula*. The CLSM micrographs show substacks of longitudinal sections. Red color indicates TTX-like immunoreactivity; green, α-acetylated tubulin immunoreactivity; blue, nuclei (DAPI). (**a**) Control sample incubated with pre-absorption anti-TTX antibodies. (**b**) Negative control section without primary antibodies an, anterior side; *bc*, buccal cavity; *cg*, cephalic gland; ds, dorsal side; *ep*, epidermis; ps, posterior side; *rc*, rhynchocoel; vs, ventral side.
